# DUPAN-II normalisation as a biological indicator during preoperative chemoradiation therapy for resectable and borderline resectable pancreatic cancer

**DOI:** 10.1186/s12885-023-10512-2

**Published:** 2023-01-18

**Authors:** Shinichiro Hasegawa, Hidenori Takahashi, Hirofumi Akita, Yosuke Mukai, Manabu Mikamori, Kei Asukai, Daisaku Yamada, Hiroshi Wada, Yoshiaki Fujii, Takahito Sugase, Masaaki Yamamoto, Tomohira Takeoka, Naoki Shinno, Hisashi Hara, Takashi Kanemura, Naotsugu Haraguchi, Junichi Nishimura, Chu Matsuda, Masayoshi Yasui, Takeshi Omori, Hiroshi Miyata, Masayuki Ohue, Osamu Ishikawa, Masato Sakon

**Affiliations:** 1 Department of Gastroenterological Surgery, Osaka International Cancer Institute, 3-1-69, Otemae, Chuo-Ku, Osaka, 541-8567 Japan; 2 Department of Gastroenterological Surgery, Graduate School of Medicine, Osaka University, 2-2, Yamadaoka, Suita, Osaka, 565-0871 Japan

**Keywords:** DUPAN-II, Pancreas, Cancer, Preoperative chemoradiation therapy

## Abstract

**Background:**

Duke pancreatic mono-clonal antigen type 2 (DUPAN-II) is a famous tumour maker for pancreatic cancer (PC) as well as carbohydrate antigen 19–9 (CA19-9). We evaluated the clinical implications of DUPAN-II levels as a biological indicator for PC during preoperative chemoradiation therapy (CRT).

**Methods:**

This retrospective analysis included data from 221 consecutive patients with resectable and borderline resectable PC at diagnosis who underwent preoperative CRT between 2008 and 2017. We focused on 73 patients with elevated pre-CRT DUPAN-II levels (> 230 U/mL; more than 1.5 times the cut-off value for the normal range). Pre- and post-CRT DUPAN-II levels and the changes in DUPAN-II ratio were measured.

**Results:**

Univariate analysis identified normalisation of DUPAN-II levels after CRT as a significant prognostic factor (hazard ratio [HR] = 2.06, confidence interval [CI] = 1.03–4.24, *p* = 0.042). Total normalisation ratio was 49% (*n* = 36). Overall survival (OS) in patients with normalised DUPAN-II levels was significantly longer than that in 73 patients with elevated levels (5-year survival, 55% vs. 21%, *p* = 0.032) and in 60 patients who underwent tumour resection (5-year survival, 59% vs. 26%, *p* = 0.039).

**Conclusion:**

Normalisation of DUPAN-II levels during preoperative CRT was a significant prognostic factor and could be an indicator to monitor treatment efficacy and predict patient prognosis.

**Supplementary Information:**

The online version contains supplementary material available at 10.1186/s12885-023-10512-2.

## Introduction

With recent advances in our understanding of the biological behaviour of pancreatic cancer (PC), the International Study Group of Pancreatic Surgery (ISGPS) and the International Association of Pancreatology (IAP) have proposed the incorporation of the serum carbohydrate antigen 19–9 (CA19-9) (a sialylated Lewis blood type antigen [1]) level as a biological factor associated with borderline resectability [2, 3]. The ISGPS definition also indicated that an elevated CA19-9 level was a biological factor associated with borderline resectable (BR) PC, even in anatomically resectable (R) PC, although this definition did not include a specific cut-off value of the CA19-9 level for borderline resectability [2]. The biological BR criteria in the IAP definition included an elevated CA19-9 level of > 500 U/mL [3]. However, this cut-off value for borderline resectability remains controversial. Nonetheless, the assessment of biological factors of PC is of clinical importance for the optimisation of treatment strategies for individual patients with PC.

The survival benefit provided by surgery alone for PC is limited [4]; thus, a multidisciplinary approach for PC is necessary to improve surgical outcomes. In this context, the significance of preoperative treatment strategies for R and BR PC has been reported [5–10]. An important unresolved issue in preoperative treatment strategies is the assessment of the efficacy of preoperative treatment. Accurate evaluation of the biological response during preoperative treatment is critical to identify the patients who truly benefit from the subsequent surgery among those with “resectable” disease (those with anatomically [surgically] resectable tumours). In previous reports, altered CA19-9 levels during preoperative treatment were significantly associated with patient outcomes after subsequent surgery; thus, this marker was considered as a clinically useful indicator to evaluate preoperative treatment efficacy [11–15].

Duke pancreatic monoclonal antigen type 2 (DUPAN-II), a precursor of CA19-9 that accumulates in the sera of patients with PC, is also a famous tumour marker of PC [16]. A limited number of reports with small patient cohorts have described the clinical significance of DUPAN-II in preoperative treatment strategies in patients with PC; however, the significance of DUPAN-II in preoperative treatment strategies for PC has not been fully investigated and remains unclear [17, 18]. Thus, the present study evaluated the clinical significance of the DUPAN-II level as a predictive indicator for the efficacy of the preoperative treatment for R and BR PC, with a focus on patients exhibiting significantly elevated DUPAN-II levels.

## Methods

### Patient population

This retrospective analysis included data from 221 consecutive patients who received preoperative gemcitabine-based CRT (G-CRT) between 2007 and 2017 at the Osaka International Cancer Institute. All patient were classified according to the Union for International Cancer Control (UICC) 8^th^ edition. Details of the eligibility criteria for our G-CRT protocol have been described previously [19]. In brief, all patients eligible for preoperative CRT were required to be treatment-naïve for PC and to exhibit the radiologic findings indicative of R and BR PC according to the classification of the National Comprehensive Cancer Network (version 1, 2017) with definite evidence of tumour extension beyond the confines of the pancreas (i.e., cStage 0 or cStage I cases according to the UICC 8^th^ edition classification were not eligible) [20]. All tumours required confirmation of invasive ductal adenocarcinoma of the pancreas based on either histological or cytological examination before the initiation of preoperative CRT.

**Fig. 1:**
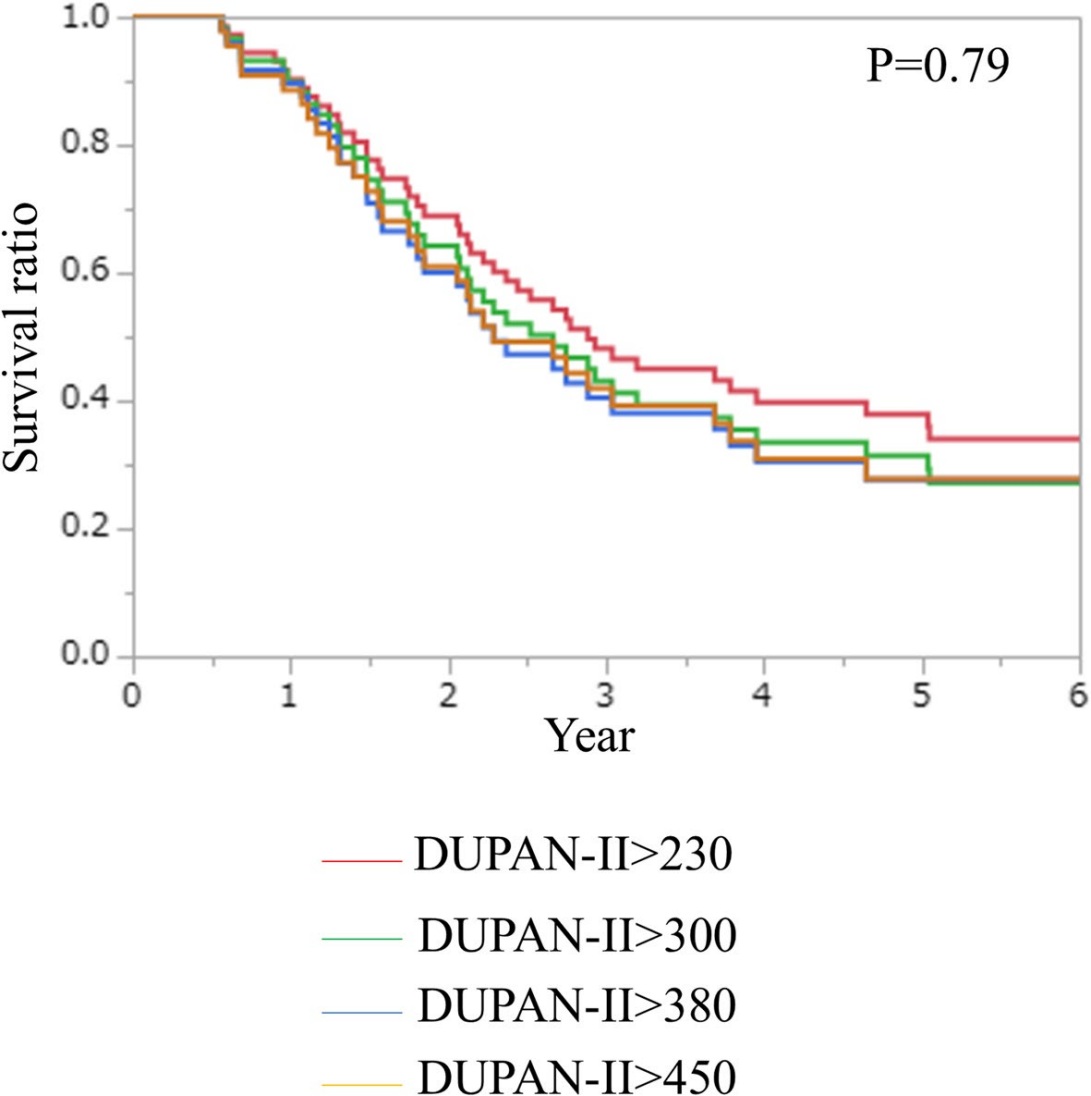
Overall survival of the patients with sufficiently elevated DUPAN-II levels, i.e. 1.5, 2, 2.5, and 3 times the upper normal limit of DUPAN-II (150 U/ml); DUPAN-II > 230, 300, 380, and 450 U/ml

This study primarily aimed to investigate the significance of the DUPAN-II level during preoperative treatment in the context of predicting oncological outcomes and monitoring preoperative treatment efficacy. Focusing on the patients with sufficiently elevated DUPAN-II levels, we configured several cut-off values of DUPAN-II levels, i.e. 1.5, 2, 2.5, and 3 times the upper normal limit of DUPAN-II (150 U/ml); DUPAN-II > 230, 300, 380, and 450 U/ml. There was no significant difference with the prognosis among those 4 cut-off values (Fig. 1). Therefore, we adopted DUPAN-II > 230 U/ml as a significantly elevated DUPAN-II level, in which the largest number of patients (*n* = 73) were included among those 4 cut-off values.

### Preoperative CRT

The details of preoperative G-CRT have been described previously [8, 19, 21]. In short, three-dimensional radiation or intensity-modulated radiation therapy (IMRT) was targeted to the following fields and administered at a total radiation dose of 50–60 Gy with a daily fraction of 2–2.4 Gy five times/week: the primary pancreatic tumour, celiac and superior mesenteric arteries, retroperitoneal soft tissue, and the para-aortic region. Intravenous administration of gemcitabine (1000 mg/m^2^) was initiated concurrently on days 1, 8, and 15 during each 4-week cycle; this procedure was repeated for three cycles, such that the preoperative CRT was completed in 3 months after initiation.

### Measurement of serum CA19-9 and DUPAN-II levels

Routine laboratory tests for CA19-9 and DUPAN-II were carried out before the initiation of preoperative therapy (pre-CA19-9 and pre-DUPAN-II) and every 4 weeks thereafter. As this study aimed to investigate whether alterations in the DUPAN-II level during preoperative CRT represented the biological response of the tumours to preoperative treatment and the consequent clinical outcomes, we assessed serum DUPAN-II levels before the initiation of preoperative treatment (pre-DUPAN-II), after the completion of preoperative treatment (post-DUPAN-II), and the proportional alteration between pre- and post-DUPAN-II levels (%DUPAN-II: log [Post DUPAN-II] / log [Pre DUPAN-II] (%)) in association with the clinicopathological factors and patient survival. Serum levels of CA19-9 and DUPAN-II were measured using chemiluminescence immunoassay (CLIA) and enzyme immunoassay (EIA) methods, respectively. Serum levels ≤ 37 U/mL for CA19-9 and ≤ 150 U/mL for DUPAN-II were considered normal in this study.

In addition to CA19-9 and DUPAN-II, we evaluated post CRT neutrophil to lymphocyte ratio (NLR) as an inflammatory marker and prognostic nutrition index (PNI) as a nutritional index, which have been reported as preoperative prognostic markers [22, 23]. We set the median value of each factor as the cut-off value.

### Surgical procedure and postoperative follow-up

Our surgical procedure consisted of pancreatectomy with extensive lymphatic and connective tissue clearance and postoperative liver perfusion chemotherapy. Additional information has been reported previously by our institute [8, 19]. In the case of unresectable PC after completion of preoperative CRT, further surgical treatment was avoided. Gastrointestinal and/or choledocointestinal bypass was performed if necessary in patients intraoperatively determined to have unresectable PC. Patients with unresectable PC were treated with clinically relevant chemotherapy. Cases with PC recurrence were administered various chemotherapy and/or radiation therapies based on the clinical indications.

Postoperative follow-up consisted of routine physical examination, radiographic imaging studies, and laboratory tests, including measurement of the serum levels of CA19-9 and DUPAN-II. Both chest X-ray and abdominal computed tomography (CT)/ultrasonography were performed every 3 months to carefully monitor for the presence or absence of cancer recurrence. Additional examinations, such as positron emission tomography/magnetic resonance imaging and cytological and histopathological examinations, were also available for the determination of PC recurrence when clinically indicated.

### Statistical analysis

Data are expressed as means ± standard deviation. The clinicopathological parameters were compared using Fisher’s exact and χ^2^ tests, while continuous variables were compared using Student’s t-tests. Overall survival was calculated from the start date of preoperative therapy to the last known date of follow-up. Survival curves were computed using the Kaplan–Meier method and differences between survival curves were compared using log-rank tests. The Cox proportional hazards regression model was used to analyse the simultaneous influence of prognostic factors. Hazard ratios (HRs) estimated from the Cox analysis were reported as relative risks with corresponding 95% confidence intervals (CIs). In all analyses, *P* < 0.05 was considered statistically significant. Statistical analysis was performed using JMP software version 13.2 (SAS Institute Inc., Cary, NC, USA).

## Results

**Fig. 2:**
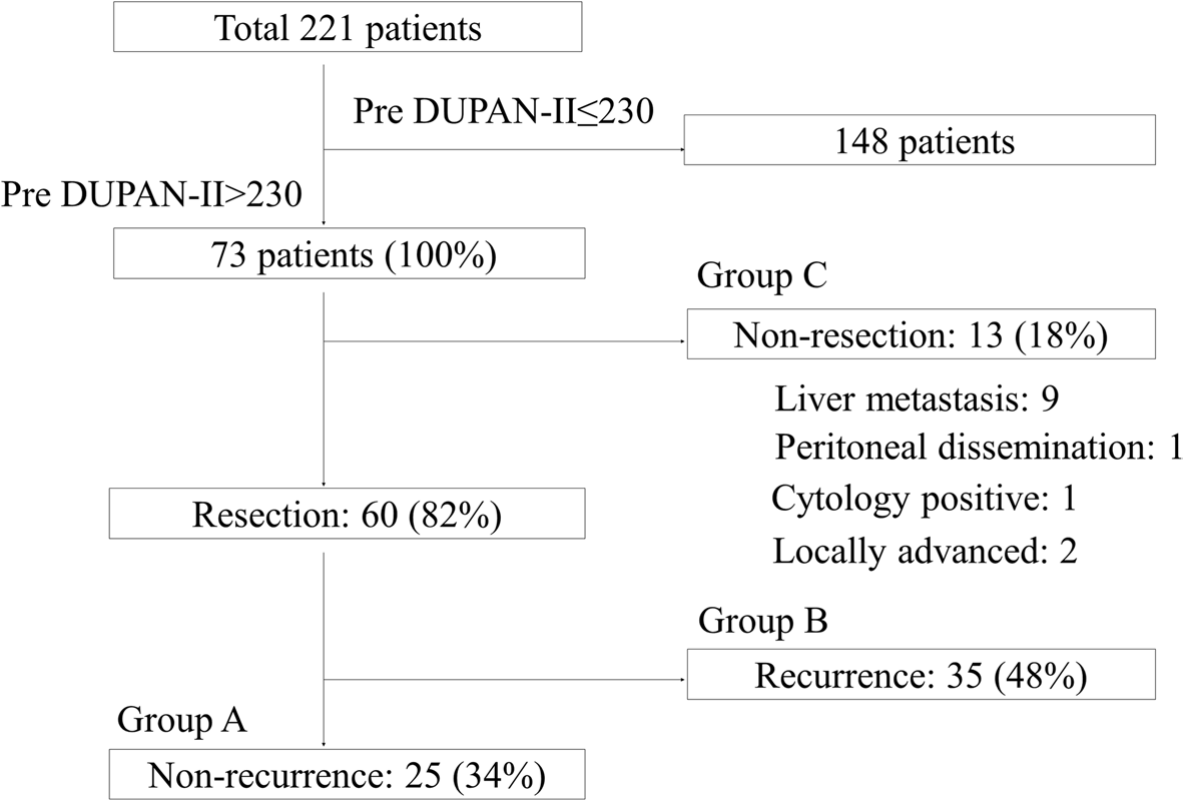
Clinical outcomes of 221 patients with pancreatic cancer (PC). The patients were divided into three groups; namely, non-recurrence (**A**), recurrence (within 5 years after the operation) (**B**), and non-resection (**C**). The reasons for non-resection are listed

In the present study, we focused on patients with significantly elevated pre-DUPAN-II level as described above (1.5 times the upper normal limit of DUPAN-II [> 230 U/mL] in our institute). Among the 221 patients included in this study, 73 had pre-DUPAN-II levels > 230 U/mL. The clinicopathological findings of these 73 patients before the initiation of preoperative CRT are summarised in Table 1. Forty and 33 patients were classified as having R and BR PC, respectively [20]. The pre-CRT DUPAN-II levels in the BR group were significantly higher than those in the R group, whereas there was no significant difference in pre-CRT CA19-9 levels between the two groups (*p* = 0.015 and 0.23, respectively). The distribution of tumour markers showed no correlation between CA19-9 and DUPAN-II (Supplementary Fig. 1). Among the 73 patients with elevated pre-CRT DUPAN-II levels, 60 (82%) underwent surgical resection after CRT and 35 (48%) experienced cancer recurrence within 5 years after surgery. The reasons for non-resection are shown in Fig. 2. Twenty-one patients (35%) were positive for lymph node metastasis in histopathological examinations. In this study, the median observation period was 33.0 (4.2–109.6) months.

**Table 1:**
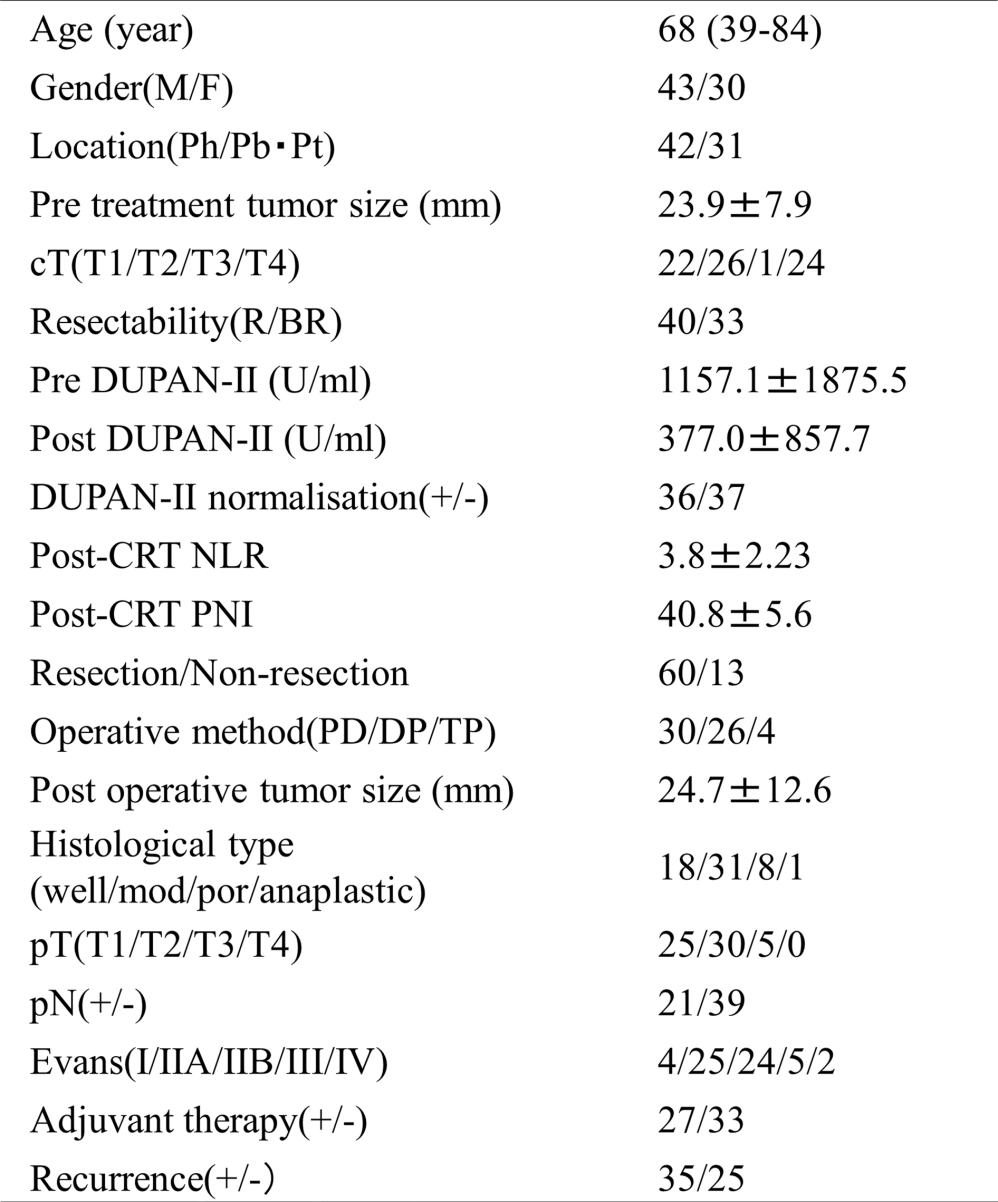
Clinicpathological findings of total 73 pancreatic (PC) Patients

c*T* clinical depth of tumor invasion, *R* resectable, *BR* borderline resectable, *pT* pathological depth of tumor invasion, *pN* pathological lumph node metastasis, *Ph* pancreas head, *Pb* pancreas body, *Pt* pancreas trail, *CRT* chemoradiation therapy, *NLR* neutrophil-to lymphocyte ratio, *PN* prognostic nutritional index, *PD* pancreatoduodenectomy, *PD* distal pancreatecomy

First, to assess the relationship between DUPAN-II levels and clinical outcomes, we divided the 73 patients into three groups: non-recurrence, recurrence (within 5 years after surgery), and non-resection (Fig. 2). The pre-CRT DUPAN-II levels in the non-recurrence group (560.3 ± 448.1 U/mL) were significantly lower than those in the recurrence group (1652.9 ± 2510.9 U/mL) (*p* = 0.018), while there was no significant difference between the non-recurrence (560.3 ± 448.1 U/mL) and non-resection (969.7 ± 1279.1 U/mL) groups (Supplementary Fig. 2, left column). The post-CRT DUPAN-II levels in the non-recurrence group (132.2 ± 124.4 U/mL) was significantly lower than those in the recurrence (460.8 ± 853.1 U/mL) and non-resection (622.5 ± 1473.7 U/mL) groups (*p* = 0.019 and 0.026) (Supplementary Fig. 2, middle column). The %DUPAN-II in the non-recurrence group (72 ± 14%) was significantly lower than that in the non-resection group (81 ± 15%) (*p* = 0.047), while there was no significant difference between the non-recurrence (72 ± 14%) and recurrence (76 ± 15%) groups (Supplementary Fig. 2, right column).

Subsequently, we assessed the clinical factors before surgery according to the postoperative overall survival among the resected cases (*n* = 60, Fig. 2). Table 2 summarises the results of these univariate and multivariate. The univariate analysis identified tumor size, cT (clinical depth of tumour invasion), resectability, post CA19-9 levels, and post DUPAN-II levels as significant prognostic factors. The multivariate analysis identified resectability as a significant and independent prognostic factor (Table 2, right column). This analysis adopted the normal upper limit of DUPAN-II (≤ 150 U/ml) and CA19-9 (≤ 37 U/ml) and the median value of %DUPAN-II (75%) as the cut-off values. The result from multivariate analysis suggested that the resectability was an extremely powerful independent prognostic factor among clinical factors. Table 3 summarises the results of the comparisons of clinicopathological factors between patients with post-CRT normalisation of DUPAN-II levels and those with persistently elevated levels. The proportions of patients with successful post-CRT DUPAN-II normalisation were 49% (36/73), including 53% (21/40) and 46% (15/33) of patients in the R and BR groups, respectively (*p* = 0.72). The overall resection rate was 82% (60/73), compared to 86% (31/36) and 78% (29/37), respectively, in patients with normalised and elevated DUPAN-II levels (*p* = 0.58).

**Table 2:**
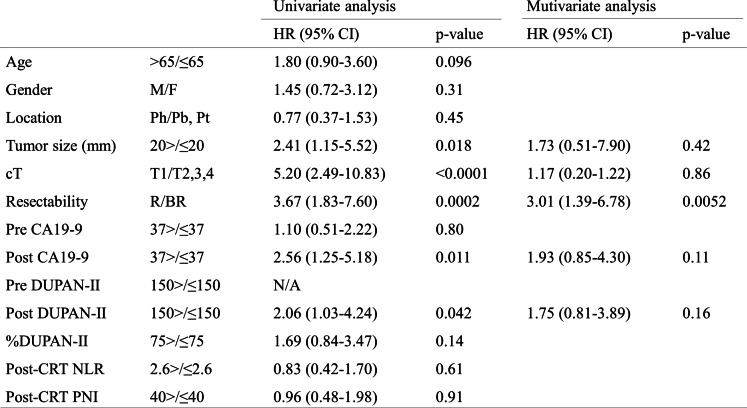
The predictive factors for the overall survival in the 60 resected cases

*Ct* clinical depth of tumor invasion, *R* resectable, *BR* borderline resectable, *Ph* pancreas head, *Pb* pancreas body, *Pt* pancreas traile, *CRT* chemoradiation therapy, *NLR* neutrophil-to-lymphocyte ratio, *PNI* prognostic nutritional index, *N/A* not applicable

**Table 3:**
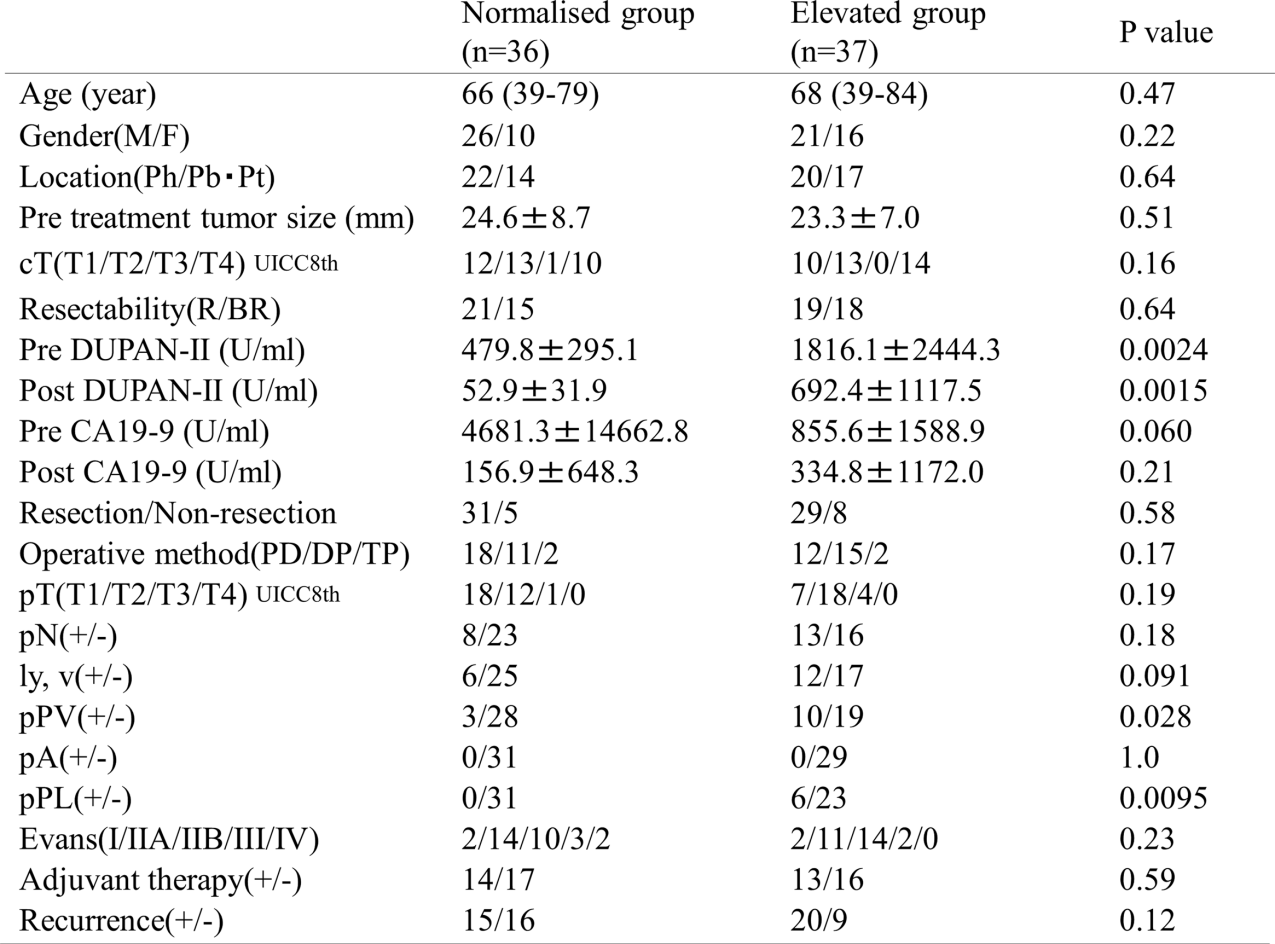
The comparison of clinicopathological factors betweew DURAN-II normalised and elevated groups

*cT* clinica depth of tumor invasion, *pT* pathological depth of tumor invasion, *pN* pathological lymph node metastasis, *Ph* pancreas head, *Pb* pancreas body, *Pt* pancreas trail, *PD* pancreatoduodenectomy, *DP* distal pancreatectomy, *TP* total pancreatectomy. *ly,v* lymph-vascular invasion, *PV* portal vain invasion, *A* arterial invasion, *PL* plexus invasion

**Fig. 3:**
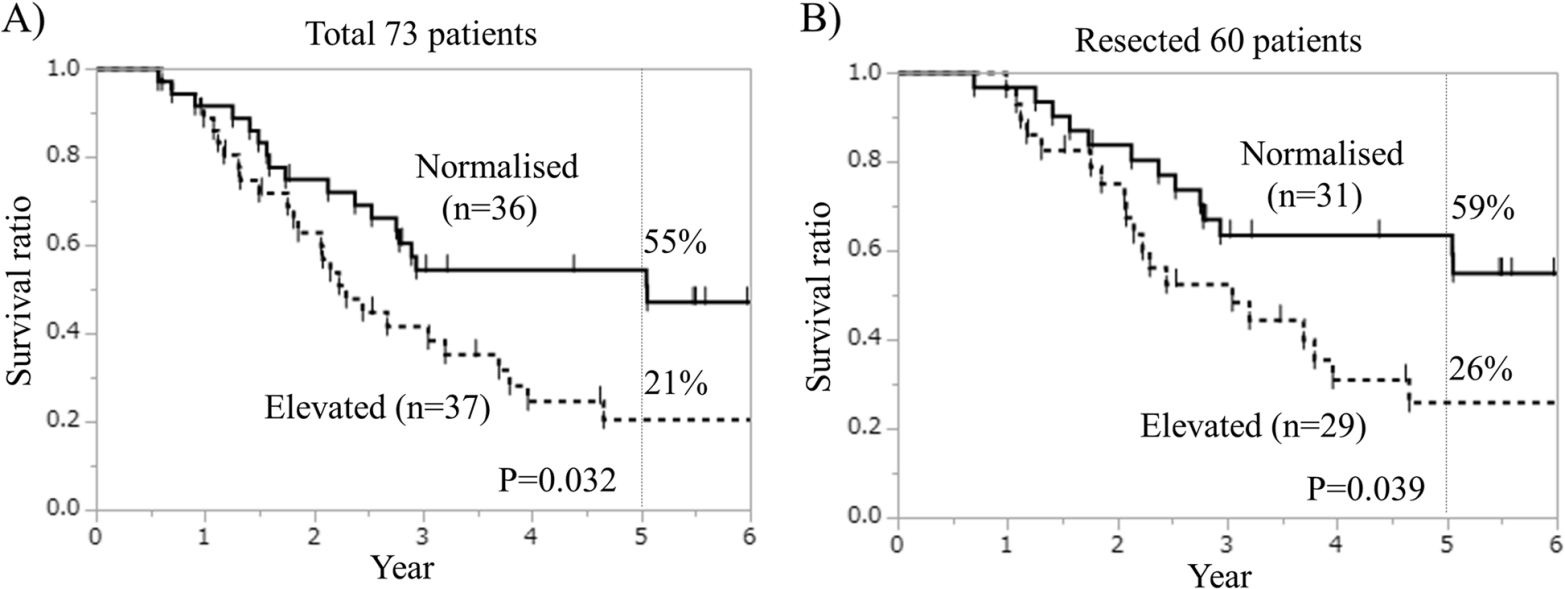
Overall survival based on the normalization of duke pancreatic monoclonal antigen type 2 (DUPAN-II) levels during preoperative chemoradiation therapy (CRT) in all 73 patients (**A**) and 60 patients who underwent resection (**B**). **C** Overall survival of patients with normalized and elevated DUPAN-II during preoperative CRT in 73 patients and those with normal pre DUPAN-II levels among all 221 patients with pancreatic cancer

Figure 3 shows the Kaplan–Meier curves for overall survival in the 73 total patients and the 60 patients who underwent resection, showing that normalisation of the DUPAN-II level was a consistent factor for favourable prognosis (*p* = 0.032 and 0.039, respectively). The 5-year survival rate of the normalised group was 55%, compared to 21% in 73 patients with elevated levels. The 5-year survival rates of the normalised and elevated groups were 59% and 26%, respectively (*p* = 0.039), among the 60 patients who underwent resection.

Among the total of 221 patients included in this study, patients with pre-DUPAN-II levels within normal limits (e.g. ≤ 150 U/mL; *n* = 127) exhibited significantly more favourable survival than those with pre-CRT DUPAN-II levels > 150 U/mL (*n* = 94), representing 5-year survival rates of 53% and 34%, respectively (*p* = 0.0026) (Supplementary Fig. 3A). On the other hand, the 5-year survival rates of the 94 patients with pre-CRT DUPAN-II levels > 150 U/ml was as low as those of the 73 patients with pre-CRT DUPAN-II levels > 230 U/ml (34% and 36%, respectively) (Supplementary Fig. 3B). However, among the 73 patients with pre-CRT DUPAN-II levels > 230 U/mL, those with post-CRT DUPAN-II normalisation showed comparable survival to patients with pre-CRT DUPAN-II levels within normal limits (Fig. 3A). Moreover, the 5-year survival rates of patients with normalised DUPAN-II levels and those with pre-CRT DUPAN-II levels ≤ 150 U/mL were 55% and 53%, respectively (*p* = 0.56, Fig. 3A and Supplementary Fig. 3A).

## Discussion

Recently, an elevated DUPAN-II level of > 2000 U/ml at diagnosis in CA19-9 non-secretors was reported to be an unfavourable prognostic factor that corresponded to an elevated CA19-9 level of > 500 U/ml in CA19-9 secretors with PC. However, it is still controversial whether DUPAN-II levels could be a predictive indicator for monitoring the efficacy of the preoperative treatment and survival in R/BR PC patients. Few reports have demonstrated the clinical implications of the DUPAN-II level during preoperative treatment for PC. Murakami et al. reported that the normalisation of serum CA19-9 or DUPAN-II levels after neoadjuvant therapy was independently associated with better overall survival in patients who underwent tumour resection [17]. However, they evaluated DUPAN-II levels in only seven patients who had undetectable CA19-9 levels. Sunagawa et al. also described DUPAN-II normalisation as a significant predictive marker for patients with PC during preoperative treatment. However, serum DUPAN-II levels before preoperative treatment were elevated in only 38 patients and normalised in only 28 patients after preoperative treatment. Therefore, it was difficult to determine the clinical significance of DUPAN-II levels in preoperative treatment strategies for PC based on these reports.

Thus, we focused on patients with significantly elevated pre-CRT DUPAN-II levels (> 230 U/ml) to evaluate the clinical significance of the DUPAN-II level in the setting of preoperative CRT for R/BR pancreatic cancer. Our results demonstrated that the normalisation of the post-CRT DUPAN-II level was a significant prognostic factor in our preoperative treatment strategy for PC, while the pre-CRT DUPAN-II level was not: the DUPAN-II normalised group had a significantly better prognosis compared to the elevated group, both in all 73 patients and the 60 patients who underwent resection (Fig. 3). In addition, only post-DUPAN-II levels among pre-, post-, and alteration of the DUPAN-II level were significantly associated with both unresectability and recurrence (Supplementary Fig. 2). Moreover, a failure to achieve post-DUPAN-II normalisation during preoperative CRT tended to be associated with a high probability of recurrence or non-resection (*p* = 0.059). Although the patients with pre-CRT DUPAN-II levels within normal limits had a significantly more favourable survival than those with DUPAN-II levels beyond the normal limit (Supplementary Fig. 3A), our findings suggest that DUPAN-II levels after the completion of preoperative treatment were more important than those before the initiation of preoperative treatment in patients with significantly elevated pre-CRT DUPAN-II levels in the setting of preoperative CRT for PC. Certainly, the surgical outcome of patients with post-CRT normalisation of DUPAN-II levels was comparably favourable to that in patients with pre-CRT DUPAN-II levels within normal limits. Considering these findings, the normalisation of DUPAN-II levels during preoperative treatment in patients with elevated pre-CRT DUPAN-II levels could be a goal of preoperative treatment for PC. Further prospective studies are needed to assess the clinical significance of the normalisation of DUPAN-II levels in the setting of preoperative treatment strategies for PC.

Focusing on the other 148 patients without significantly elevated DUPAN-II levels (pre-DUPAN-II ≤ 230), the post-DUPAN-II levels of 144 patients were within normal limits and the prognosis of them was pretty good (5-year survival rate, 50%) (Supplementary Fig. 4). Even in those patients without relevant elevated DUPAN-II levels, post-DUPAN-II levels could be a prognostic factor, however, not to be an indicator to monitor preoperative treatment efficacy for PC. The distribution of tumour markers was classified into four groups, with both pre-CA19-9 and DUPAN-II elevated, only pre-CA19-9 elevated, only pre-DUPAN-II elevated, and both pre-CA19-9 and DUPAN-II within normal limits (Supplementary Fig. 5). Evaluating the change of tumour markers in each group before the initiation of preoperative CRT, DUPAN-II normalisation could be achieved in 47 (65%) (21 [9%] and 26 [36%]) patients of the 72 with pre-CA19-9 and DUPAN-II elevated and in 7 (32%) patients of the 22 patients with only pre-DUPAN-II elevated. DUPAN-II elevation was observed in only 1 patient of the 127 with pre-DUPAN-II normalised through preoperative CRT.

The management of patients without post-CRT normalisation of DUPAN-II levels also clinically important and should be addressed based on the findings of the current study. Although the failure of post-CRT DUPAN-II normalisation was associated with a higher probability of unresectability, recurrence, and impaired survival even in resected cases, the 5-year survival rate of the resected cases was as high as 26% (Fig. 3). Considering that 5-year survival in patients with PC without surgical intervention is quite rare, the failure of DUPAN-II normalisation post-CRT alone does not necessarily preclude surgical resection. However, these patients are at a high risk of recurrence and impaired prognosis. These findings suggest that prolonged preoperative treatment with repeated radiographic evaluations might be useful in identifying patients who are more likely to benefit from subsequent surgery. Alternatively, adjuvant treatment with prolonged duration and/or more potent regimens might improve the surgical outcomes in patients with failure of post-CRT DUPAN-II normalisation. Further investigation is needed to establish in the preoperative treatment strategy the optimal clinical management of patients who do not achieve DUPAN-II normalisation post-CRT.

This study had several limitations. The data were collected at a single institute and retrospectively analysed; thus, care should be taken when interpreting these results owing to potential biases. Moreover, post-operative liver perfusion chemotherapy is not so common. However, we adopt this method as an adjuvant chemotherapy for PC and have reported the effectiveness of liver perfusion chemotherapy for pancreatic cancer [24, 25]. In addition, we did not evaluate the significance of DUPAN-II in association with the CA19-9 level and the presence/absence of Lewis antigen because of the relatively small number of patients in this study. However, our results warrant further investigations to test the clinical significance of DUPAN-II in preoperative treatment strategies. Multi-institutional prospective studies including larger numbers of patients are needed to verify our findings.

In conclusion, normalisation of the DUPAN-II level during preoperative CRT was a significant prognostic factor in patients with R/BR PC. Therefore, the assessment of DUPAN-II levels could be an indicator to monitor treatment and predict consequent prognosis in certain clinical situations.

## Supplementary Information

**Additional file 1.**

**Additional file 2.**

**Additional file 3.**

**Additional file 4.**

**Additional file 5.**

## Data Availability

The data that support the findings of this study are available on request from the corresponding author. The data are not publicly available due to privacy or ethical restrictions.
