# Supplementary figures and images for: DUPAN-II normalisation as a biological indicator during preoperative chemoradiation therapy for resectable and borderline resectable pancreatic cancer
Source: BMC Cancer. 2023 Jan 18;23:63. doi: 10.1186/s12885-023-10512-2 (PMC9850710; doi:10.1186/s12885-023-10512-2)

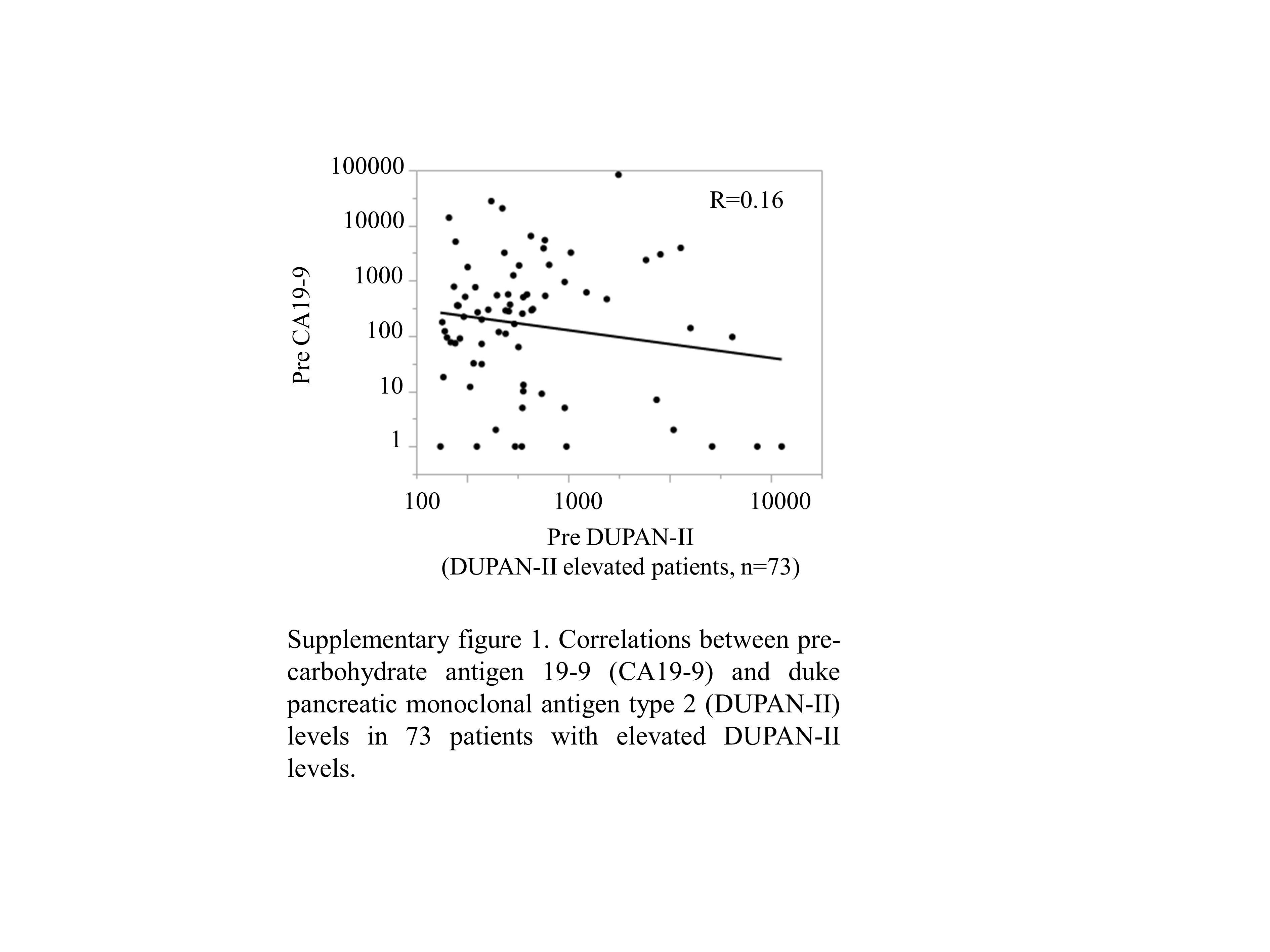

Supplement: Supplementary file 1 — Additional file 1. [file 12885_2023_10512_MOESM1_ESM.tif]

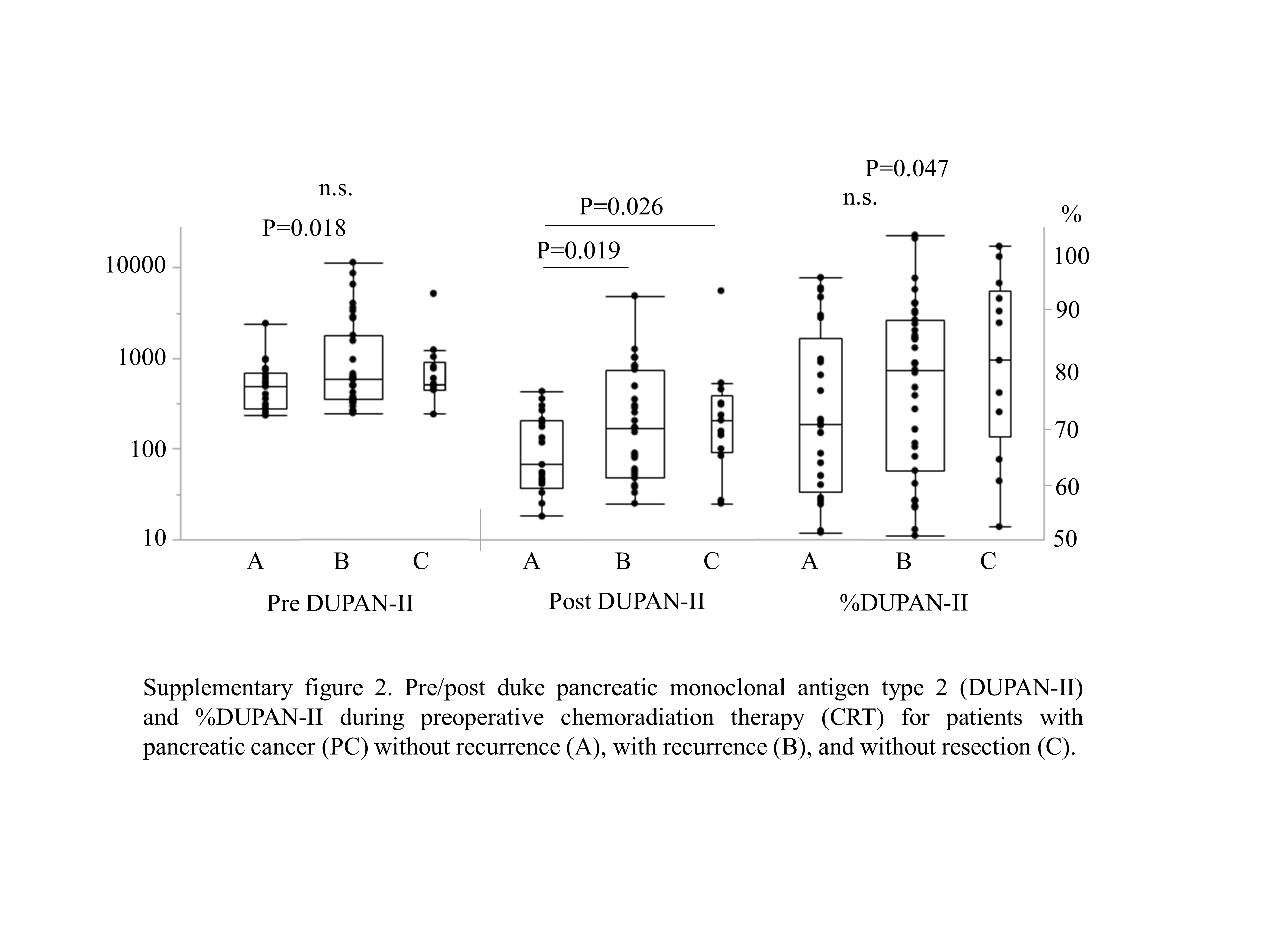

Supplement: Supplementary file 2 — Additional file 2. [file 12885_2023_10512_MOESM2_ESM.tif]

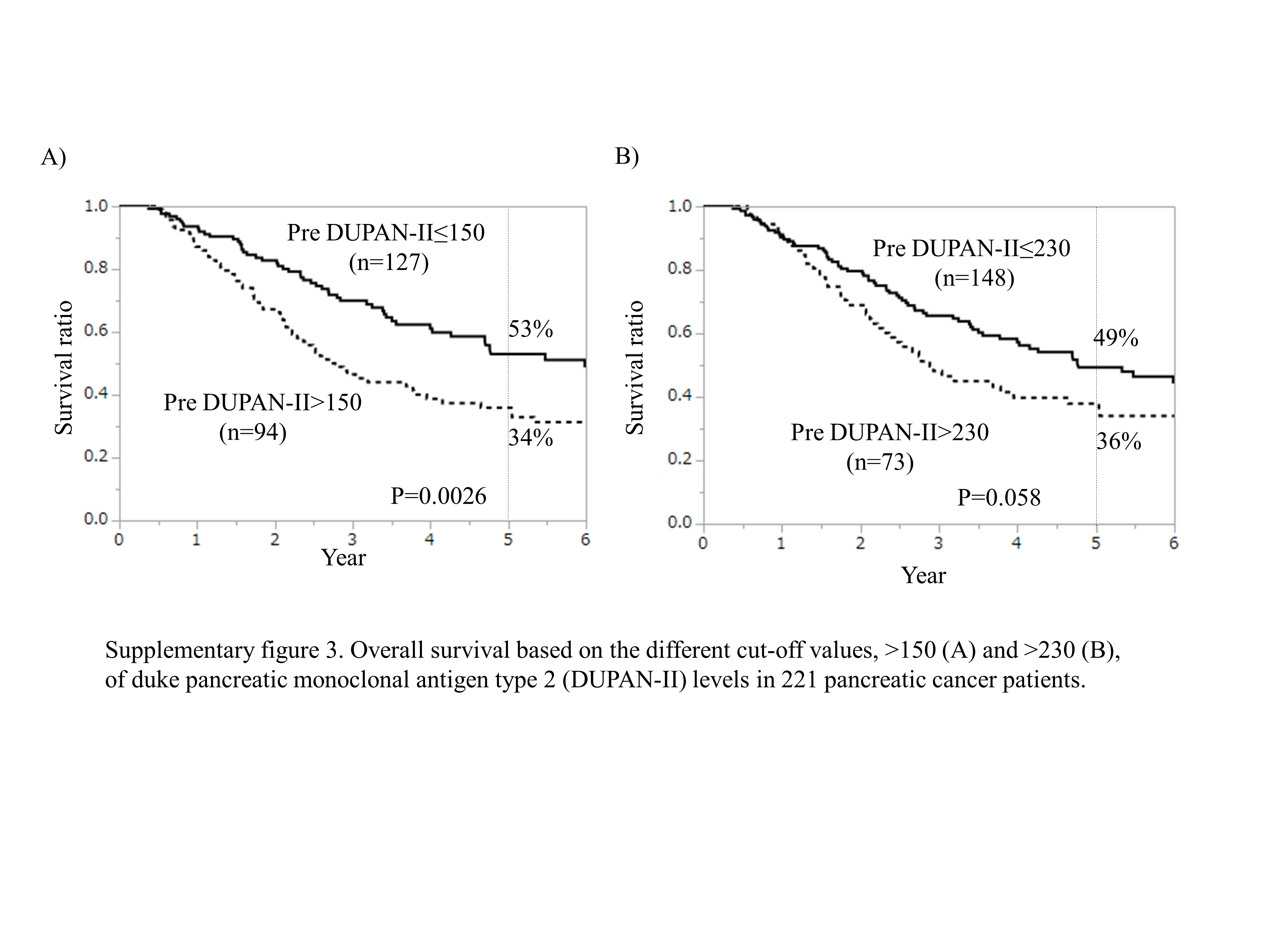

Supplement: Supplementary file 3 — Additional file 3. [file 12885_2023_10512_MOESM3_ESM.tif]

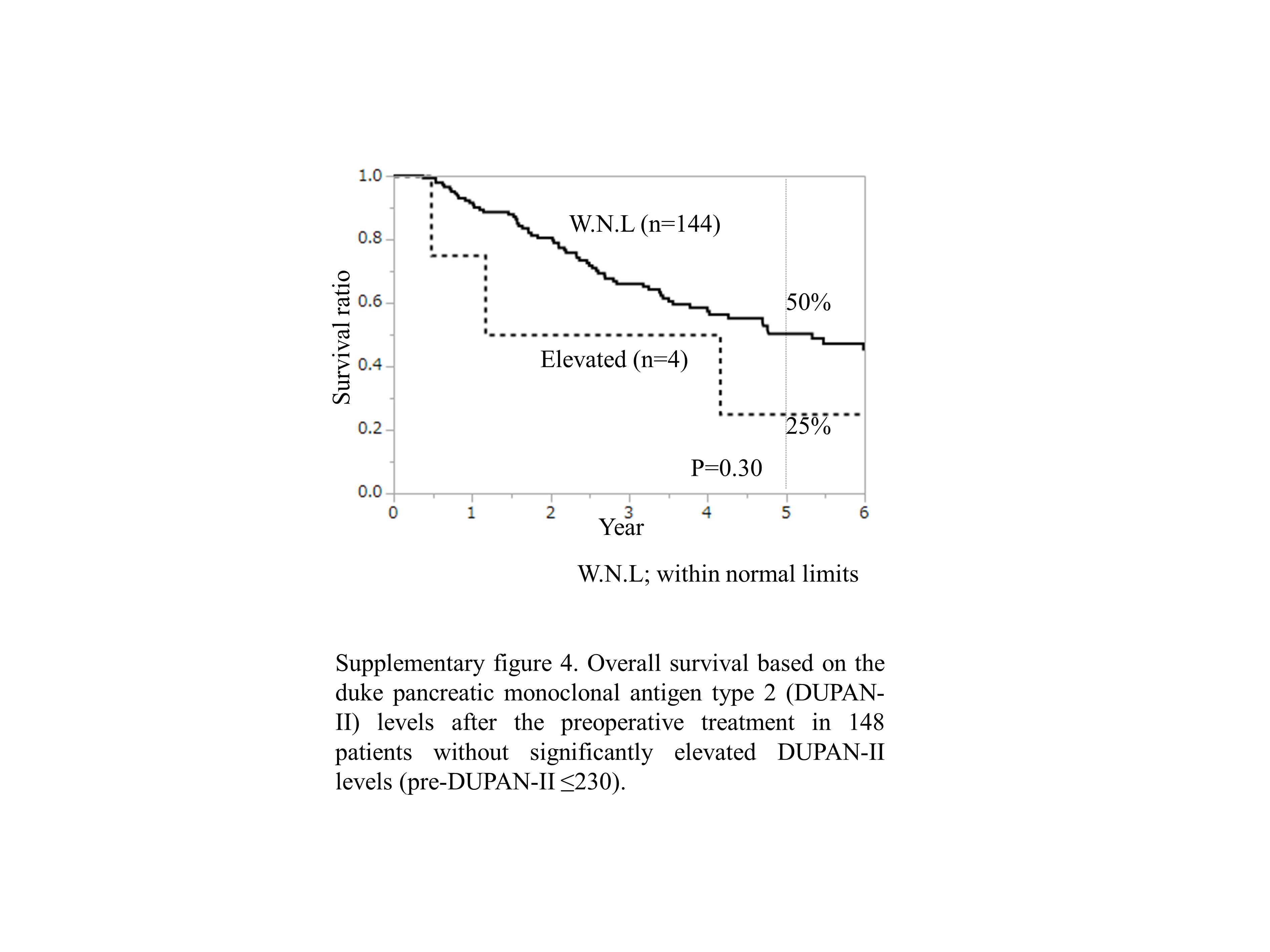

Supplement: Supplementary file 4 — Additional file 4. [file 12885_2023_10512_MOESM4_ESM.tif]

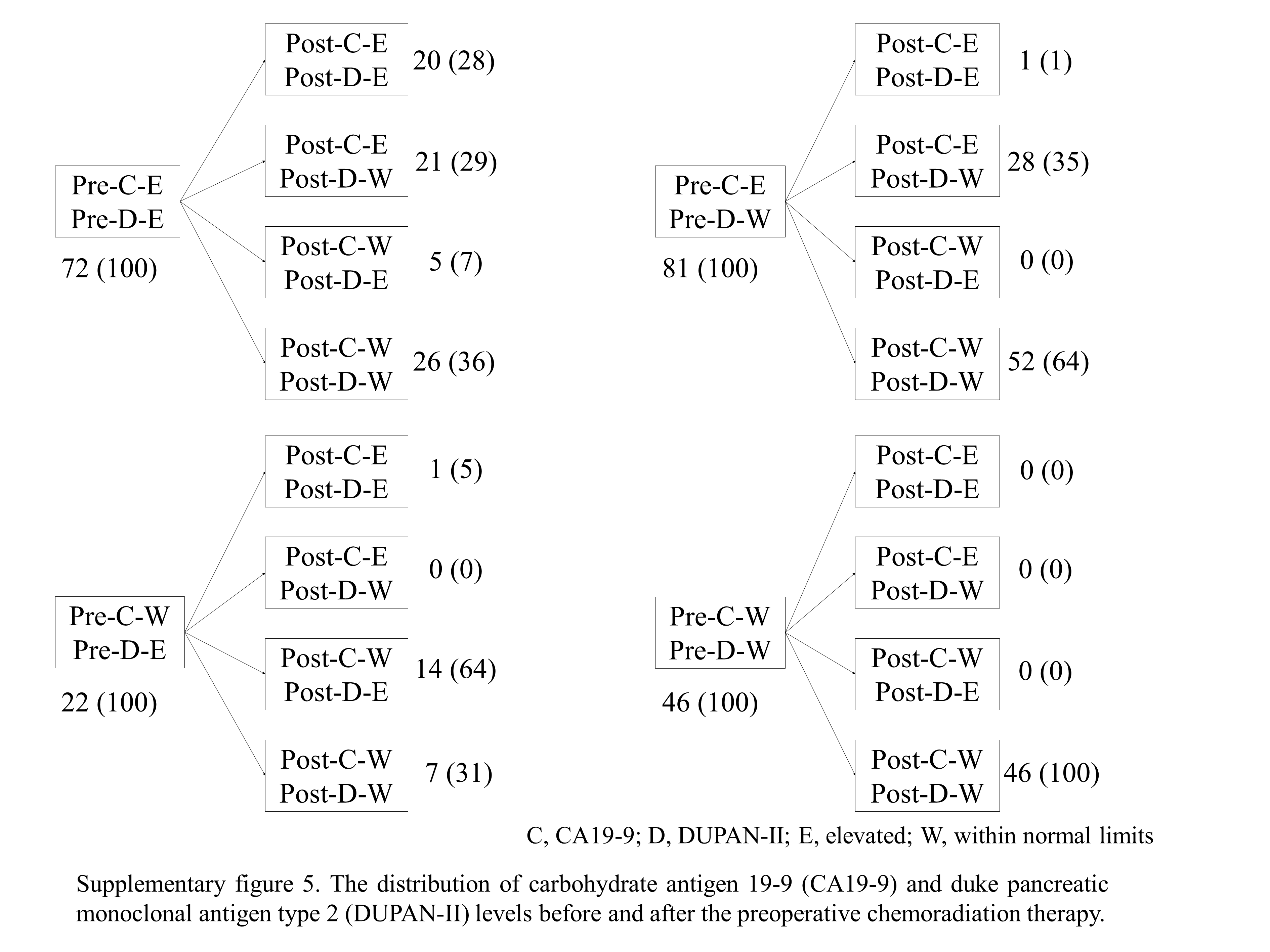

Supplement: Supplementary file 5 — Additional file 5. [file 12885_2023_10512_MOESM5_ESM.tif]
